# *ASNEO*: Identification of personalized alternative splicing based neoantigens with RNA-seq

**DOI:** 10.18632/aging.103516

**Published:** 2020-07-22

**Authors:** Zhanbing Zhang, Chi Zhou, Lihua Tang, Yukang Gong, Zhiting Wei, Gongchen Zhang, Feng Wang, Qi Liu, Jing Yu

**Affiliations:** 1 Department of Ophthalmology, Shanghai Tenth People’s Hospital, Tongji University, School of Medicine, Tongji University, Shanghai 200009, China; 2 Department of Endocrinology and Metabolism, Shanghai Tenth People's Hospital, Bioinformatics Department, School of Life Sciences and Technology, Tongji University, Shanghai 200009, China; 3 Department of Gastroenterology, Shanghai Tenth People's Hospital; Bioinformatics Department, School of Life Sciences and Technology, Tongji University, Shanghai 200009, China; 4 School of Geographic and Biologic Information, Nanjing University of Posts and Telecommunications, Nanjing 210023, China

**Keywords:** cancer, neoantigen, immunotherapy, alternative splicing, RNA-seq

## Abstract

Cancer neoantigens have shown great potential in immunotherapy, while current software focuses on identifying neoantigens which are derived from SNVs, indels or gene fusions. Alternative splicing widely occurs in tumor samples and it has been proven to contribute to the generation of candidate neoantigens. Here we present *ASNEO*, which is an integrated computational pipeline for the identification of personalized Alternative Splicing based NEOantigens with RNA-seq. Our analyses showed that *ASNEO* could identify neopeptides which are presented by MHC I complex through mass spectrometry data validation. When *ASNEO* was applied to two immunotherapy-treated cohorts, we found that alternative splicing based neopeptides generally have a higher immune score than that of somatic neopeptides and alternative splicing based neopeptides could be a marker to predict patient survival pattern. Our identification of alternative splicing derived neopeptides would contribute to a more complete understanding of the tumor immune landscape. Prediction of patient-specific alternative splicing neopeptides has the potential to contribute to the development of personalized cancer vaccines.

## INTRODUCTION

Cancer neoantigens have shown great potential in targeted immunotherapies due to their immunogenicity and lack of expression in normal tissues, which could be recognized by autologous T cells and thus may constitute the Achilles heel of tumor cells. Previous studies have demonstrated that somatic DNA alterations, e.g. nonsynonymous point mutations, insertion-deletions (indels), gene fusions and/or frameshift mutations, are the sources of neoantigens, which have potential pathogenic impact on gene expression, protein function and downstream pathways [1]. Several tools have been developed to identify these kinds of neopeptides including *pVAC-Seq* [2], *MuPeXI* [3], *TSNAD* [4], *CloudNeo* [5], *INTEGRATE-neo* [6], *Neopepsee* [7] and *pTuneos* [8], while transcriptome in tumors received much less attention. Recent studies showed that transcriptome level modifications could also be a potential source of neopeptides by analyses of TCGA cohorts [9] as well as the clinical cohorts undergo immune checkpoint inhibitor therapy [10]. Kahles et al. analyzed the alternative splicing (AS) across 32 The Cancer Genome Atlas (TCGA) cancer types from 8,705 patients and detected alternative splicing events and tumor variants by reanalyzing RNA and whole-exome sequencing data [9]. Smart et al. analyzed intron retention events with immunotherapy treated cohorts and identified neopeptides derived from them [10]. These studies both indicated that transcriptome level modifications could generate neopeptides, which were supported by mass spectrometry (MS) analysis, and they proposed that AS neopeptides should be considered for prospective personalized cancer vaccine development. However, an efficient and easy-to-use tool is still lacking to accurately predict and investigate the personalized AS neopeptides from transcriptome. In this study, we present *ASNEO*, an integrated computational pipeline for the identification and investigation of personalized Alternative Splicing based NEOantigens from RNA-seq. It is an efficient tool to identify alternative splicing based neopeptides and can be quickly installed and deployed at https://github.com/bm2-lab/ASNEO.

To further investigate the features of AS neopeptides, we applied *ASNEO* to two published immunotherapy-treated cohorts. We investigated the difference between AS neopeptides and somatic neopeptides, and evaluated the correlations between AS neopeptides and patient immunotherapy response or patient survival time. Our comprehensive analyses indicated that (1) alternative splicing based neopeptides generally have a higher immune score than that of somatic neopeptides, demonstrating that alternative splicing based neopeptides might be a better candidate as the cancer vaccine, and (2) alternative splicing based neopeptides might be a marker to predict patient survival pattern.

## RESULTS

### General workflow for integrated analysis

**Figure 1:**
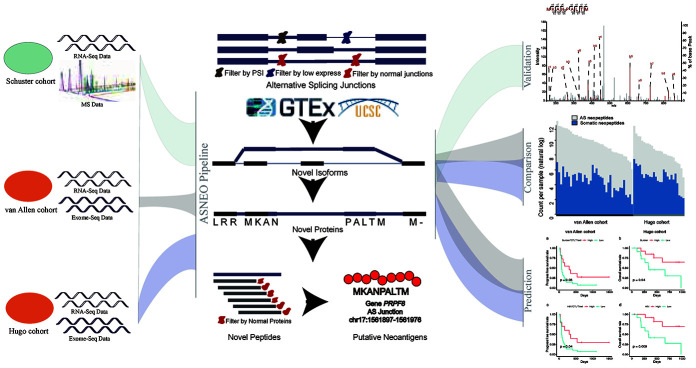
**A comprehensive workflow to analyze AS neopeptides by *ASNEO*.** The left part represents the data of three cohort used in the analyses. The middle part represents the computational pipeline of *ASNEO* for identification of AS neopeptides. The right part represents three main analyses performed in this work, containing evaluation of *ASNEO* by mass spectrometry (MS) analysis (top), immune score comparison of AS neopeptides with somatic neopeptides (middle), and predictability evaluation of AS neopeptides by survival analysis (bottom).

We presented a versatile and comprehensive workflow to analyze AS neopeptides by *ASNEO* (Figure 1). In our study, three cohorts were used to identify AS neopeptides by *ASNEO*, which is presented as an integrated computational pipeline for the identification of personalized Alternative Splicing based NEOantigens with RNA-seq. Three main analyses were performed, including evaluation of the performance of *ASNEO*, immunogenicity comparison of AS neopeptides with somatic neopeptides, and predictability evaluation of response or survival with AS neopeptides.

### Computational pipeline of *ASNEO*

**Figure 2:**
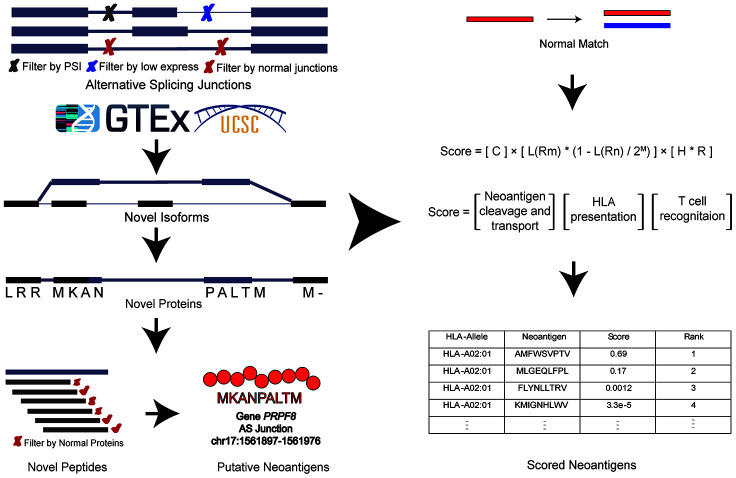
**The computational pipeline of *ASNEO* for identification of AS neopeptides.** The *ASNEO* accepted splice junctions detected by *STAR*, and then filtered low expressed junctions, low psi junctions, and *Normal Junctions*. Next, *ASNEO* inserted the filtered junctions into reference isoforms to generate novel isoforms and translated the novel isoforms to novel proteins by one-frame translation from the translation start site to the stop codon. The filtered novel proteins then were chopped up to 8-11-mer peptides, which were filtered by *Normal Proteins*. The bind ranks of remained peptides to HLA were calculated by *NetMHCpan-4.0* and those peptides whose %rank<2 were considered as putative neopeptides. In addition, *ASNEO* integrated an immune score to evaluate the immunogenicity of putative neopeptides with several features, including the mutant peptide-MHC %rank, the normal peptide-MHC %rank, the number of mismatches between the mutant peptide and normal peptide as well as the cleavage probability, the TAP transport efficiency, the hydrophobicity score and the T cell recognition probability of mutant peptide.

The general computational framework of *ASNEO* consists of the following steps (Figure 2). (1) RNA-seq reads were cleaned and aligned to reference genome hg19, generating splice junctions along with the predicted HLA types, which were taken as the input to *ASNEO*. (2) *ASNEO* filtered low expressed junctions and *Normal Junctions*. The *Normal Junctions* were defined as those junctions with at least 2 reads covered by at least 1% of normal samples (>3000) from GTEx detected junctions, as well as those junctions in the UCSC hg19 reference annotation. (3) The filtered novel junctions were inserted into reference isoforms to generate novel isoforms. Then these isoforms were translated into novel proteins by one-frame translation from the translation start site to the stop codon. In this way, proteins whose length>30 are retained. (4) *ASNEO* chopped up the proteins into 9-mer peptides and these peptides were filtered by the set of *Normal Proteins*. In our study, *Normal Proteins* consist of UCSC reference proteins and *Normal Junctions* produced proteins by our pipeline. (5) To calculate the bind rank of peptides to HLA, *NetMHCpan-4.0* was used and those rank<2% peptides are considered as putative neopeptides. (6) In addition, *ASNEO* designed an immune score schema to evaluate the immunogenicity of putative neopeptides with several features including mutant peptide-MHC %rank, the normal peptide-MHC %rank, the number of mismatches between the mutant peptide and normal peptide, the peptide cleavage probability, TAP transport efficiency, hydrophobicity score and T cell recognition score, which has been proposed in our previous study [11].

### Evaluation of *ASNEO*

To evaluate the prioritization performance of *ASNEO*, our previous study [11] has already applied the score schema to five public peptides datasets with experimentally confirmed immunogenic and non-immunogenic peptides. Compared to other available tools, including the neoantigen *fitness model* [12], *MuPeXI* [3], *neopepsee* [7] and a tool available at IEDB [13], in 2 of 5 peptides datasets, our score scheme presented the highest ROC-AUC (area under the receiver operating characteristic curve) and in 3 of 5 peptides datasets, our score scheme presented the highest PR-AUC (area under the precision-recall curve), indicating the superiority and rationality of our proposed score schema.

**Figure 3:**
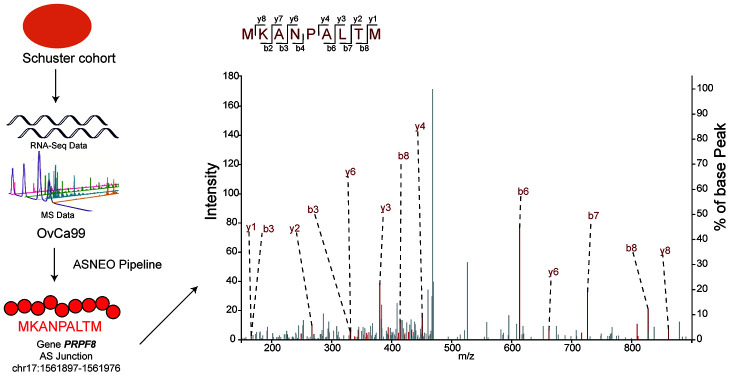
**Predicted AS neopeptides from Schuster cohort were identified by mass spectrometry bound to MHC class I.** The AS neopeptide MKANPALTM identified in the Schuster cohort originating from gene *PRPF8* (chr17:1561897-1561976) was predicted by *ASNEO* and validated by mass spectrometry in OvCa99 immunopeptidome.

To check the reliability of *ASNEO*, mass spectrometry analysis was performed on Schuster cohort [14]. *ASNEO* was applied to RNA-seq data to identify putative AS neopeptides, and only peptides with 9-mer length and percentile %rank<2 were retained. *ASNEO* identified an average of 69.8 AS neopeptides per sample, range from 6 to 194. The total number of unique neopeptides of all 14 samples was 407, which account for less than half of the number of total neopeptides. Mass spectrometry data was applied to search against the database consisting of *Normal Proteins* plus the putative AS neopeptides by each sample. To guarantee a high confidence, a 1% false discovery rate was set. As a result, the predicted AS neopeptides MKANPALTM from sample OvCa99 and IHFLSLLNF from sample OvCa48 were experimentally discovered in complex with MHC I via mass spectrometry with a high confidence (Figure 3). The score of MKANPALTM was 1.9e-07 while the score of IHFLSLLNF was 7.31e-07, rank 208/407 and 187/407 relatively. The discovery of peptides existing in both mass spectrometry data and computational predicted AS neopeptides indicating that *ASNEO* can identify AS neopeptides effectively, which were processed and presented through the MHC I pathway.

### Comparison of AS neopeptides to somatic neopeptides in immunotherapy-treated cohorts

**Figure 4:**
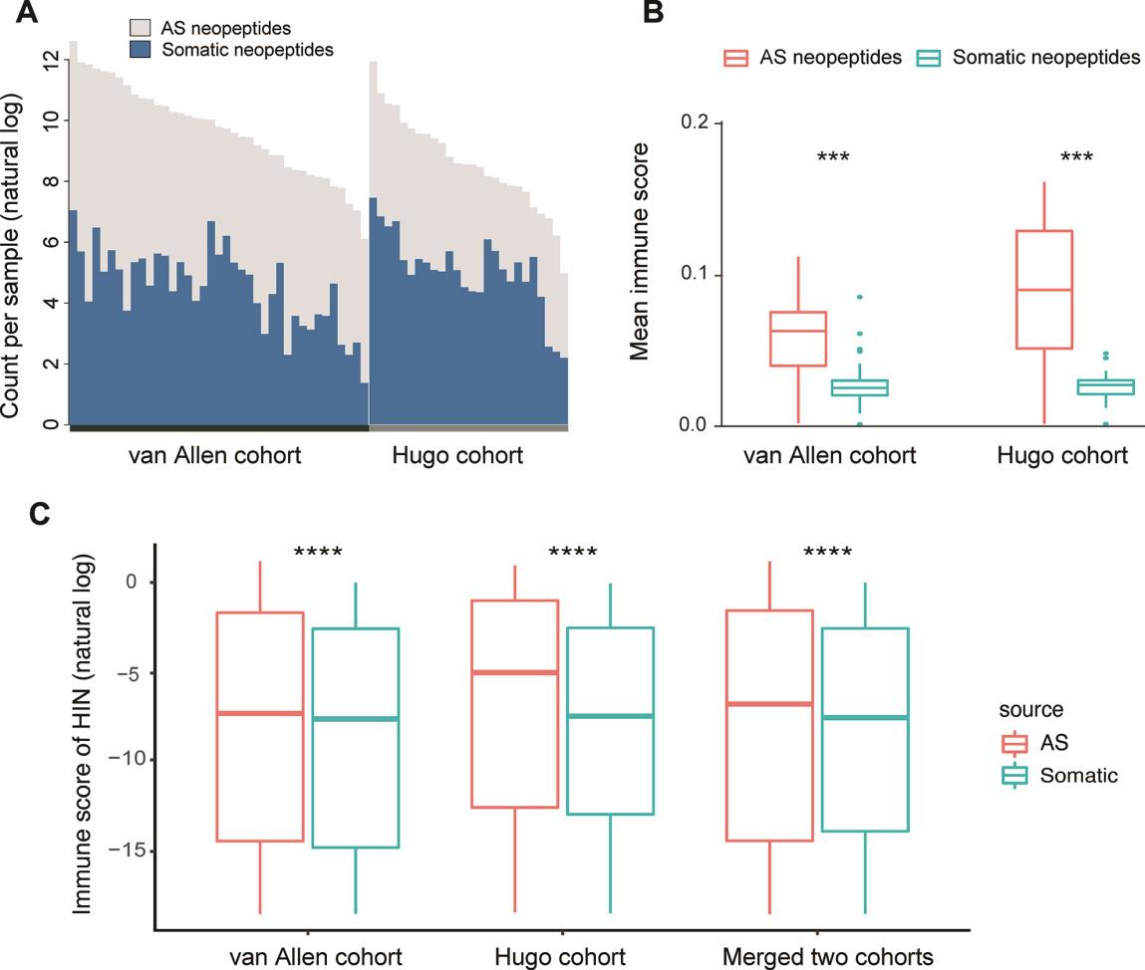
**Comparison of AS neopeptides with somatic neopeptides.** (**A**) The number of somatic neopeptides and AS neopeptides. Within each cohort, patients were sorted by total neoantigen burden. Neopeptide counts (y-axis values) were represented in natural log format. (**B**) In each cohort, AS neopeptides showed a higher mean immune score profile than that of somatic neopeptides (van Allen cohort: Wilcoxon test P=1.5e-08; Hugo cohort: Wilcoxon test P=9.1e-06). (**C**) Using HIN, AS neopeptides showed a significant higher immune score than that of somatic neopeptides in both van Allen cohort and Hugo cohort as well as in merged two cohorts (Wilcoxon test, P<2.2e-16 for all). The immune scores of neopeptides (y-axis values) were represented in natural log format. HIN: high immune neopeptides.

To assess the characteristics of AS neopeptides, we applied *ASNEO* to two published datasets of melanoma patients treated with CTLA4 inhibitors [15] or PD-1 inhibitors [16]. For van Allen cohort, in the 39 melanoma patients, we identified an average of 301.7 putative AS neopeptides per sample, range from 24 to 2406 (Supplementary Table 1). For Hugo cohort, in the 25 melanoma patients, we identified an average of 47.6 AS putative neopeptides per sample, range from 5 to 121 (Supplementary Table 2). To compare the AS neopeptides with somatic neopeptides, the whole exome sequencing (WES) data in van Allen cohort and Hugo cohort were applied to *MuPeXI* to predict somatic neopeptides, which were then scored by *ASNEO*. We identified an average of 186.9 (range from 4 to 1160) somatic neopeptides in van Allen cohort (Supplementary Table 1) and an average of 293.7 (range from 9 to 1733) somatic neopeptides in Hugo cohort (Supplementary Table 2). For van Allen cohort and Hugo cohort, the total neopeptides yield a ~1.6-fold increase and a ~0.16-fold increase respectively with the addition of AS neopeptides (Figure 4A).

The mean score profile, which is defined by the mean of immune scores for all identified neopeptides in a sample, was compared. The result indicated that AS neopeptides showed a higher mean score profile than that of somatic neopeptides in both van Allen cohort (Wilcoxon test, P=1.5e-08) and Hugo cohort (Wilcoxon test, P=9.1e-06) (Figure 4B). However, when the median score profile, which is defined by the median of immune scores for all identified neopeptides in a sample, was compared, the result showed no difference between AS neopeptides and somatic neopeptides neither in van Allen cohort nor in Hugo cohort (Wilcoxon test, p<0.05 for both). Therefore, we defined those neopeptides whose immune score greater than 1e-8 as the high immunogenicity neopeptides (HIN). The cutoff of 1e-8 was set due to its approaching to the median value of all neopeptides.

When comparing the scores of AS neopeptides with somatic neopeptides, we found that the scores of AS neopeptides were higher in van Allen cohort (Wilcoxon test, p=4e-11), but not in Hugo cohort (Wilcoxon test, p=0.47). While using the HIN, AS neopeptides had a significant higher immune score than that of somatic neopeptides both in van Allen cohort and in Hugo cohort as well as in merged two cohorts (Wilcoxon test, P<2.2e-16 for all) (Figure 4C), indicating that AS neopeptides might be more immunogenic, could be a better candidate as cancer vaccines.

### Prediction of response or survival with AS neopeptides in immunotherapy-treated cohorts

**Figure 5:**
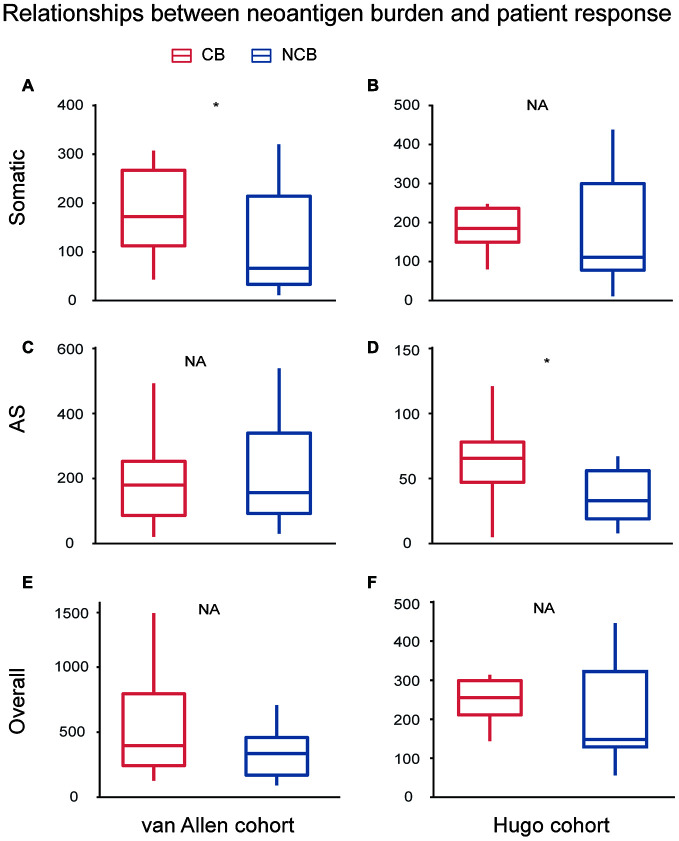
**Relationships between neoantigen burden and patient response undergo immunotherapy treatment.** For van Allen cohort, the somatic neoantigen burden was significant associated with patient response to immunotherapy treatment (Wilcoxon test, P=0.031, **A**), while AS neoantigen burden was not associated with response (Wilcoxon test, P=0.93, **C**). For Hugo cohort, the somatic neoantigen burden was not associated with response (Wilcoxon test, P=0.37, **B**), while AS neoantigen burden was significant associated with response (Wilcox test, P=0.029, **D**). Overall neoantigen burden showed no association with response neither in van Allen cohort (Wilcox test, P=0.24, **E**) nor in Hugo cohort (Wilcox test, P=0.2, **F**). CB: Clinical Benefit, NCB: No Clinical Benefit.

To investigate the relationship between the checkpoint inhibitor response and neoantigen burden, including somatic neoantigen burden and AS neoantigen burden, van Allen cohort and Hugo cohort were compared between two clinical response groups: clinical benefit and no clinical benefit respectively. For van Allen cohort, the somatic neoantigen burden was significant associated with patient response to immunotherapy treatment (Wilcoxon test, P=0.031, Figure 5A), consistent with previous study [15], but AS neoantigen burden was not associated with the response (Wilcoxon test, P=0.93, Figure 5C). For Hugo cohort, the somatic neoantigen burden was not associated with response (Wilcoxon test, P=0.37, Figure 5B), consistent with previous study as well [16], while AS neoantigen burden was significant associated with response (Wilcoxon test, P=0.029, Figure 5D). The results suggested that AS neopeptides could predict response in certain cases, just like somatic neopeptides, but when or where need to be further investigated. Then we combined the somatic neopeptides and AS neopeptides together, termed as the overall neopeptides, and assumed that the overall neoantigen burden of samples derived from all sources have association with response in all cases. However, the analysis showed that there was no association between overall neopeptides burden and response neither in van Allen cohort nor in Hugo cohort (Wilcoxon test, van Allen cohort: P=0.24, Hugo cohort: P=0.2, Figure 5E, 5F). Additionally, we explored whether there was correlation between AS neoantigen burden and somatic neoantigen burden or expression of canonical markers of immune cytolytic activity CD8A, GZMA or PRF1 [17]. The results showed there was no association between AS neoantigen burden and somatic neoantigen burden, nor association between AS neoantigen burden and CD8A, GZMA or PRF1 neither in van Allen cohort nor in Hugo cohort (Pearson correlation P>0.05 for all).

**Figure 6:**
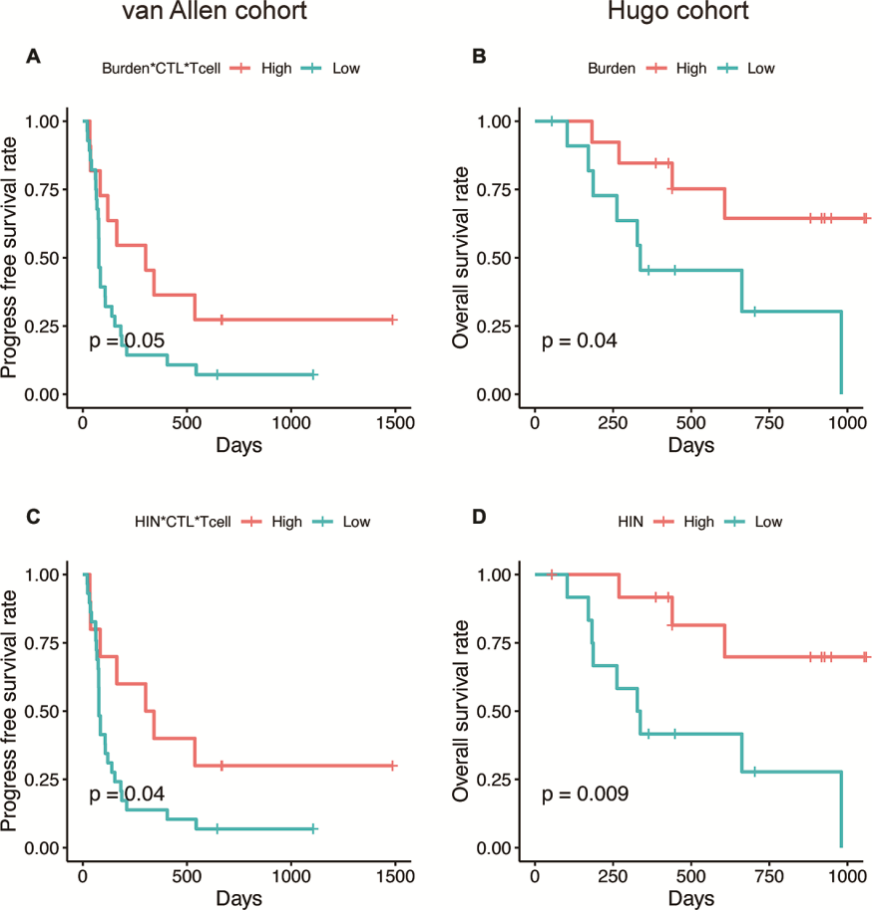
**The AS neopeptide is a potential biomarker for patient survival analysis.** Neoantigen burden multiplying CTL and T cell abundance (Burden*CTL*Tcell) was associated with progress free survival in van Allen cohort (log-rank P=0.05, **A**) and neoantigen burden (Burden) was associated with improved overall survival (log-rank P=0.04, **B**) in Hugo cohort. When use high immune neopeptides (HIN) to predict survival, the separation became more significant in both van Allen cohort (HIN*CTL*Tcell, log-rank P=0.04, **C**) and Hugo cohort (HIN, log-rank P=0.009, **D**).

Next, we explored whether the AS neoantigen burden could predict patient survival. We firstly investigated the expression level of TGFB1 in these two cohorts, which is a suppressor of cytotoxic lymphocyte (CTL) [18]. We found that the TGFB1 is highly expressed in Hugo cohort rather than van Allen cohort (Wilcoxon test, P=0.04), which indicated that the tumor microenvironment differences should be considered in these two cohorts. Hence, the AS neoantigen burden multiplying CTL and T cell abundance (Burden*CTL*Tcell) was defined as a metric to predict the patient survival in van Allen cohort while in Hugo cohort only neoantigen burden was considered. Here, we examined whether the expression level of TGFB1, T cell abundance or CTL abundance possess the survival predictive power or not. Our analysis results showed that the abundance of TGFB1, CTL, T cell or CTL*Tcell were not associated with patients' survival neither in van Allen cohort nor in Hugo cohort (log-rank test, P<0.05 for all, Supplementary Figure 1). But the AS neoantigen burden multiplying CTL and T cell abundance was associated with progress free survival (PFS) in van Allen cohort (log-rank P=0.05, Figure 6A) and neoantigen burden was associated with improved overall survival (OS) in Hugo cohort (log-rank P=0.04, Figure 6B). When using the HIN instead of neoantigen burden as a predictor, the survival separations became more significant both in van Allen cohort (HIN*CTL*Tcell, log-rank P=0.04, Figure 6C) and in Hugo cohort (HIN, log-rank P=0.009, Figure 6D), which suggested that the HIN could be a better biomarker. In addition, we examined the metrics (van Allen cohort: Burden*CTL*Tcell, HIN*CTL*Tcell; Hugo cohort: Burden, HIN) together with gender and age in multivariable Cox regression analysis, and the results indicated that all these metrics were associated with the patients' survival, independent of gender and age (Supplementary Table 3).

## DISCUSSION

Alternative splicing events have been shown contribution to cancer development and progression and potential to generate neoantigens [9, 10]. In this work, we presented *ASNEO*, an integrated computational pipeline for the identification of personalized Alternative Splicing based NEOantigens from RNA-seq. Identification of alternative splicing derived neoantigens will contribute to a more complete understanding of the tumor immune landscape. Prediction of patient-specific AS neoantigens has the potential to contribute to the development of personalized cancer vaccines. In our comprehensive analyses, taking together, *ASNEO* was demonstrated to be an efficient in silico prediction tool for identifying and prioritizing cancer neopeptides derived from alternative splicing. It adopts several preliminary filtering strategies to obtain reliable neopeptides; integrates a neoantigen scoring schema to evaluate the immunogenicity of putative neopeptides for neoantigens prioritizing; and implements multiple thread processing for running speed acceleration. We validated the reasonability of *ASNEO* by applying it to an independent dataset containing both RNA-seq data and MS data. The neopeptides MKANPALTM and IHFLSLLNF were not only identified by *ASNEO* but also experimentally discovered in complex with MHC I via mass spectrometry with high confidence. Though the percentage of neopeptides validated is 0.49% that seems to represent a low proportion, it was in the range of other proteogenomics studies which also report less than 1% validation rate. For instance, Bassani-Sternberg et al. [19] identified 11 out of 3487 somatic mutations (0.32%) and Zhang et al. [20] identified 3 out of 1369 RNA editing sites (0.21%). This divergence might be influenced by LC-MS sensitivity and biological factor such as proteasome processing, cytosolic peptidases, TAP and binding affinity to HLA. We further applied *ASNEO* to two clinical cohorts undergoing checkpoint blockade immunotherapy, and revealed that AS neopeptides generally have a higher immunogenicity than somatic neopeptides, and might be a better candidate as the cancer vaccine. We also demonstrated that AS neopeptides could be a predictor of patient survival pattern. In summary, the identification of AS neopeptides not only extends the scope of neoantigen types, providing a better choice of cancer vaccine candidate, but also presents a biomarker for patient survival prediction in the context of tumor immunotherapy.

Identification of a wide array of tumor neoantigens, including those derived from SNVs, indels, gene fusions, aberrant gene expression and alternative splicing, will contribute to a more complete understanding of the tumor immune landscape. The relationships between the neoantigen burden of different sources as well as between the neoantigens and the effects of immunotherapy require further study. Currently, our evaluations are limited by the datasets with both RNA-seq and mass spectroscopy data of eluted epitopes, by the availability of public neoantigens confirmed processed and presented in vivo or not. Also, our findings are limited by the availability of clinically annotated cohorts with high-quality RNA-seq and matched normal tissue. Future development of *ASNEO* will include three main aspects: (1) Incorporation of matched normal tissue that represent normal gene expression for increasing precision of our filtering approach. (2) Incorporation of mass spectroscopy data processing into the pipeline for further filtering of neoantigen candidates. (3) Investigation of MHC-II bind peptide identification and evaluation.

## MATERIALS AND METHODS

### Design of *ASNEO*

The general computational framework of *ASNEO* consists of the following steps (Figure 1): (1) Raw RNA-seq reads were cleaned and aligned to human reference genome (hg19) to generate splice junctions along with the predicted patient-specific HLA alleles, which were taken as the input to *ASNEO*. (2) *ASNEO* filtered low expressed junctions, low psi junctions and *Normal Junctions*. The *Normal Junctions* were defined as those junctions with at least 2 reads covered by at least 1% (~30) of normal samples from GTEx [21] detected junctions, as well as those junctions in the UCSC hg19 reference annotation. (3) The filtered novel junctions were inserted into reference isoforms to generate novel isoforms. Then these isoforms were translated into novel proteins by one-frame translation from the translation start site to the stop codon. Here we adopted one-frame translation but not three-frame translation or six-frame translation to keep a lower false positive rate. In this way, proteins whose length>30 are retained. (4) *ASNEO* chopped up the proteins into 8-11-mer peptides and these peptides were filtered by the set of *Normal Proteins*. In our study, *Normal Proteins* consist of UCSC reference proteins and *Normal Junctions* produced proteins by our pipeline. (5) *ASNEO* used *NetMHCpan* version 4.0 [22] to calculate the bind rank of peptides to MHC-I, and those %rank below the threshold value (default 2) peptides are considered as putative neopeptides, the threshold value can be defined by users. (6) Additionally, to prioritize neopeptides, *ASNEO* implemented an immune score schema to evaluate the immunogenicity of putative neopeptides with several features, including the mutant peptide-MHC %rank, the normal peptide-MHC %rank, the mismatch base-pairs between the mutant peptide and normal peptide as well as the cleavage probability, the TAP transport efficiency, the hydrophobicity score and the T cell recognition probability of mutant peptide. As note, this score schema has been proposed in our previous study [11].

### Processing of RNA-seq data

RNA-seq raw data were cleaned by *Trimmomatic-0.36* [23] with standard adapters trimmed and low quality reads filtered as following: LEADING:20 TRAILING:15 SLIDINGWINDOW:4:15 MINLEN:20. Then the trimmed reads were aligned to reference human genome hg19 by *STAR* version 2.5 [24] with the UCSC RefSeq (refGene) annotation of hg19, which was downloaded from UCSC Table Brower [25]. Here we chose *STAR* to identify splice junctions to be consistent with the choice of *rMATS* [26]. The detailed parameters were setting as following:

STAR --genomeDir GNEOME --readFilesIn READ1 [READ2] --runThreadN 20 –outFilterMultimapScoreRange 1 --outFilterMultimapNmax 20 –outFilterMismatchNmax 10 --alignIntronMax 500000 –alignMatesGapMax 1000000 --sjdbScore 2 –alignSJDBoverhangMin 1 --genomeLoad NoSharedMemory –outFilterMatchNminOverLread 0.33 –outFilterScoreMinOverLread 0.33 --sjdbOverhang 100 --outSAMstrandField intronMotif –sjdbGTFfile RefSeq.gtf

Then a BAM file was sorted and indexed by *samtools* [27]. HLA alleles of each sample were inferred from trimmed data using *OptiType* [28] with default settings, which could achieve HLA typing with ~97% accuracy.

### Identification of putative neopeptides

The splice junctions detected by *STAR*, BAM file generated by *samtools* and HLA alleles predicted by *OptiType* were taken as input to *ASNEO* together. *ASNEO* adopted several steps to identify putative neopeptides from alternative splicing junctions, showing as follows:

1. *ASNEO* adopted several preliminary strategies to filter the splice junctions.
  - The unique mapped reads of junctions should greater than 10 (default setting).
  - The psi5 and psi3 should greater than 0.1 (default setting). The psi5 means how often is this donor site (5’ splice site) used with this acceptor site (3’ splice site), compared to all other acceptors. As the same, psi3 means how often is this acceptor site used with this donor site, compared to all other donors. Psi5 and psi3 are calculated by *sj2psi*, a python package.
  - Filter splice *Normal Junctions*. The *Normal Junctions* were defined as those junctions with at least 2 reads detected in at least 1% (~30) of normal samples from GTEx detected junctions, as well as those junctions in the UCSC hg19 reference annotation. To reduce likely false positive junctions, the ‘panel of normals’ approach was taken in an attempt to filter out splice junctions commonly retained in normal samples, which would not produce immunogenic peptides as a result of likely host immune tolerance.
2. *ASNEO* accepted the filtered splice junctions to generate novel isoforms, which referred to *QUILTS* [29] and *rMATS* [26]. For one junction, *ASNEO* performed the following steps:
  - Find all mapped isoforms.
  - Filter the isoforms. For an isoform, if the junction is annotated (junction exists in the isoform), tag 0; novel junction (junction not exist while both donor site and acceptor site exist), tag 1; novel donor site or novel acceptor site; tag 2; both sites are novel, tag 3. Then retain the isoforms with the smallest tag and calculated RPKM value>1.
  - Insert the junction into the retained isoforms if the junction is in the protein coding region and novel exon modified length is within 2-250bp as well as novel intron has length>50, the parameters were set the same as those of *rMATS* [26]. These settings were targeted to obtain isoforms in line with the actual biological phenomenon as far as possible.
3. *ASNEO* translated novel isoforms to proteins with one-frame translation from the translation start site to the stop codon, and proteins whose length greater than 30 were retained.
4. To generate novel peptides, remained proteins were chopping up into 8-11-mer peptides and these peptides were filtered by *Normal Proteins*. In our study, *Normal Proteins* consist of UCSC reference proteins and *Normal Junctions* produced proteins by our pipeline.
5. *ASNEO* used *NetMHCpan* version 4.0 to calculate the percentile bind ranks of peptides to patient-specific HLA alleles and those %rank<2 (default setting) peptides were considered putative neopeptides.

### Scoring of putative neoantigen

*ASNEO* designed an immune score to evaluate the immunogenicity of putative neopeptides with several features, including the mutant peptide-MHC %rank, the normal peptide-MHC %rank, the number of mismatches between the mutant peptide and normal peptide as well as the cleavage probability, the TAP transport efficiency, the hydrophobicity score and the T cell recognition score of mutant peptide, which were based on our previous work [11].

*R_m_*: The %rank affinity of the candidate neoantigen, as output by *NetMHCpan*.

*R_n_*: The %rank affinity of the normal peptide, as output by *NetMHCpan*. The normal peptide was defined as a single peptide the same long with candidate neoantigen and most similar to it in the *Normal Proteins*.

*M*: The number of mismatches between the candidate neoantigen and normal peptide.

*C*: The combined score of binding affinity, proteasomal C terminal cleavage and TAP transport efficiency of candidate neoantigen, as output by *NetCTLpan*.

*H*: The hydrophobicity score for the candidate neoantigen, determined by a machine-learning model using peptide hydrophobicity information.

*R*: The T cell recognition probability of the candidate neoantigen peptide-MHC complex, which was presented as *fitness score* [12].

The immune score ***S*** in our study was defined as:

$$S=\left[\;C\;\right]\;\times \left[L\left(R_{m}\right)*\left(1-\frac{L\left(R_{n}\right)}{2^{M}}\right)\right]\;\times \;\left[\;H*R\;\right]\;$$ (1)

Where L(x) is a logistic function given by:

$$L(x)=\frac{1}{1+e^{5(x-2)}}$$ (2)

It should be note that our previous work proposed the score schema to evaluate the immunogenic potential of the gene fusion based neopeptides. But all the factors relevant to the immunogenic potential in score scheme is only peptide specific, so it can be employed to evaluate the AS based candidate neopeptides as well.

### Collection of clinical cohort data

Three cohorts were collected and applied in our analyses, including Schuster cohort [14], van Allen cohort [15] and Hugo cohort [16]. Schuster cohort contained RNA-seq data (accession: PRJNA398141) and mass spectrometry (MS) data (accession: PXD007635), which were used to validate our *ASNEO* by MS analysis. In our study, we selected 14 patients of serous ovarian carcinoma with corresponding 14 RNA-seq data and 66 MHC-I mass spectrometry data for further validation. Van Allen cohort and Hugo cohort contained melanoma patients treated with immune checkpoint inhibitors, which had both RNA-seq data and WES data. RNA-seq data were used to identify AS neopeptides while WES data were used to identify somatic neopeptides. The van Allen cohort (accession: phs000452.v2.p1), contained 42 patients treated with anti-CTLA-4 antibodies with high-quality RNA-seq data. In our analysis, 3 patient samples were excluded due to irregular RNA sequencing size (Pat41) or lacking of corresponding high-quality WES data (Pat20 and Pat91). The Hugo cohort (accession: GSE78220) contained 27 patients treated with anti-PD1 antibodies and 2 samples were excluded because of repeating samples (Pt27) or lacking of overall survival (OS) information (Pt8). The response data and survival data of immunotherapy-treated patients in van Allen cohort and Hugo cohort were retrieved from original studies. For van Allen cohort, patients were classified into 3 groups: Response, Non-response and Long-survival while for Hugo cohort, patients were classified into PD (Progressive Disease), PR (Partial Response) and CR (Complete Response).

### Evaluation of *ASNEO*

We applied *ASNEO* to Schuster cohort, which contains 14 serous ovarian carcinoma patients with 14 RNA-seq data and 66 mass spectrometry data of MHC-I complex available. RNA-seq data were applied to *ASNEO* to identify putative neopeptides with only 9-mer length and a threshold of %rank<2. The *msCovert* [30] was used to convert raw MS data to mzML format, which following search against the database consisting of *Normal Proteins* plus the putative neopeptides from each sample by *Comet* [31] search engine. Mass tolerance for processing was 5 ppm for precursor ions and 0.5 Da for fragment ions. No cleavage specificity was selected and the only dynamic modification allowed was oxidized methionine. All the parameters we utilized here were retrieved from the original study [14]. Peptide confidence was determined using *Percolator* [32] algorithm with a target value of 1% false discovery rate. As a result, the neopeptides MKANPALTM and IHFLSLLNF were discovered and visualized by *xiSPEC* [33], an interactive tool for visualizing and analyzing mass spectrometry data.

### Identification of AS neopeptides and somatic neopeptides in immunotherapy-treated cohorts

RNA-seq data in van Allen cohort and Hugo cohort were used to identify neopeptides derived from alternative splicing by *ASNEO* with default settings, while the whole exome sequencing (WES) data were utilized to predict somatic neopeptides by *MuPeXI*. For van Allen cohort, VCF file was generated by the following analyses: WES data were trimmed by Trimmomatic [23]. Then the reads were aligned to hg38 using the Burrows-Wheeler Aligner [34], a BAM file was sorted and produced with the Picard version 2.3.0 SortSam, and duplicate reads were marked and removed using the Picard tool MarkDuplicates. Base recalibration was performed with GATK version 3.8.0 [35] to reduce false-positive variant calls. SNV calls and indel calls were performed with GATK Mutect2. For Hugo cohort, VCF file was retrieved from the original study. Kallisto [36] was utilized to quantify the abundance of gene isoforms from the RNA-seq data with the reference transcriptome downloaded from the Ensembl database for GRCh38 using Ensembl genome browser version 89 [37]. HLA alleles were obtained from *ASNEO* pipeline. Then the VCF file, gene abundance file and HLA alleles were input to *MuPeXI* to predict somatic neopeptides. Here we only identified 9-mer length somatic neopeptides, being consistent with AS neopeptides identified by *ASNEO*. The putative somatic neopeptides were further scored using our *ASNEO* score schema.

### Prediction of response or survival with AS neopeptides in immunotherapy-treated cohorts

Van Allen cohort and Hugo cohort were classified into two clinical response groups: clinical benefit and no clinical benefit. For van Allen cohort (n=39), response (n=13) samples were regarded as clinical benefit samples, while nonresponse (n=21) samples were regarded as no clinical benefit samples. The long-survival group was ignored. For Hugo cohort (n=25), PD (Progressive Disease, n=13) group was treated as no clinical benefit while PR (Partial Response, n=10) and CR (Complete Response, n=2) groups were treated as clinical benefit. In our study, we used Wilcoxon rank sum test to determine the difference of neoantigen burden between the clinical benefit group and no clinical benefit group.

The survival analysis was performed by *R* package survival and survminer and log-rank test was used to assess the correlation between survival and metrics. Mean value was used to classify the samples to high and low categories with different metrics such as neoantigen burden, HIN burden, Burden*CTL*Tcell and HIN* CTL*Tcell. HIN was defined as high immunogenicity neopeptides (HIN), which represented those neopeptides whose immune score greater than 1e-8. T cell abundance and CTL (cytotoxic lymphocyte) abundance were calculated by *MCPcounter* [38], which is an R package to predict the abundance of 10 cell populations (8 immune populations, endothelial cells and fibroblasts) from transcriptomic profiles of human tissues.

## Supplementary Material

Supplementary Figure 1

Supplementary Table 1

Supplementary Table 2

Supplementary Table 3
