# Supplementary material for: ASNEO: Identification of personalized alternative splicing based neoantigens with RNA-seq
Source: Aging (Albany NY). 2020 Jul 22;12(14):14633–48. doi: 10.18632/aging.103516 (PMC7425491; doi:10.18632/aging.103516)
Supplement: Supplementary Figure 1 [file aging-12-103516-s004..pdf]

SUPPLEMENTARY FIGURE

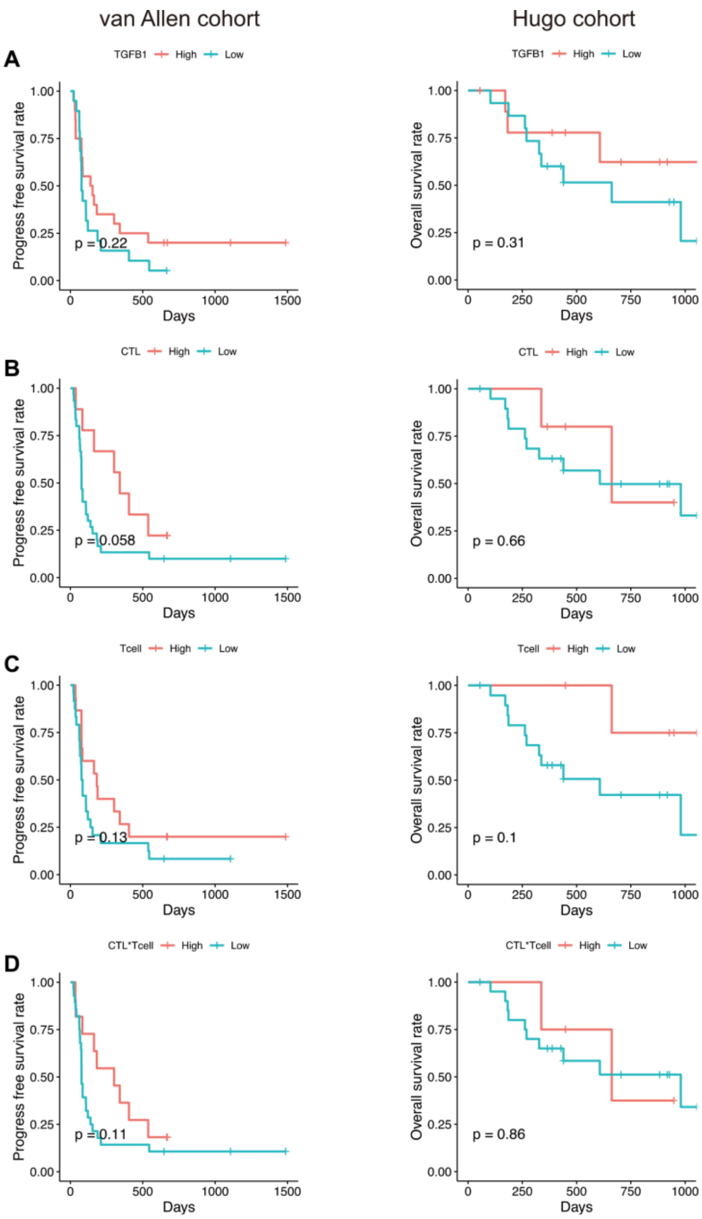

Supplementary Figure 1. Survival analysis with different metrics. (**A**) The TGFB1 abundance could not separate patients in both cohorts. (**B**) The CTL abundance could not separate patients in both cohorts. (**C**) The T cell abundance could not separate patients in both cohorts. (**D**) The CTL*Tcell abundance could not separate patients in both cohorts.
