# Supplementary material for: ASNEO: Identification of personalized alternative splicing based neoantigens with RNA-seq
Source: Aging (Albany NY). 2020 Jul 22;12(14):14633–48. doi: 10.18632/aging.103516 (PMC7425491; doi:10.18632/aging.103516)
Supplement: Supplementary Table 1 [file aging-12-103516-s002..docx]

**Supplementary Table 1. Information about 39 patients used in van Allen cohort.**

| Patient | Sample | Response | PFS | Event | T cell | CTL | AS_N | AS_HIN |
| --- | --- | --- | --- | --- | --- | --- | --- | --- |
| pat28 | SRR2660032 | long-survival | 77 | 1 | 5.195519769 | 3.139361333 | 130 | 59 |
| pat81 | SRR2661579 | nonresponse | 76 | 1 | 19.59186283 | 0.958996 | 119 | 71 |
| pat39 | SRR2665516 | response | 1487 | 0 | 5.927518125 | 4.491694 | 2406 | 1208 |
| pat15 | SRR2669446 | nonresponse | 31 | 1 | 0.14865325 | 0.519090667 | 132 | 77 |
| pat80 | SRR2674135 | response | 182 | 1 | 11.83207521 | 5.4715695 | 90 | 45 |
| pat126 | SRR2675767 | response | 187 | 1 | 8.24155475 | 4.132315667 | 242 | 99 |
| pat29 | SRR2681702 | response | 341 | 1 | 7.536742929 | 22.50492467 | 91 | 44 |
| pat02 | SRR2689710 | response | 538 | 1 | 4.362513545 | 40.8738998 | 457 | 213 |
| pat03 | SRR2689711 | nonresponse | 76 | 1 | 2.360177583 | 5.578332167 | 542 | 252 |
| pat50 | SRR2700736 | nonresponse | 65 | 1 | 4.448761111 | 4.2709342 | 343 | 131 |
| pat88 | SRR2712420 | response | 665 | 0 | 6.263597143 | 11.577765 | 183 | 94 |
| pat40 | SRR2751521 | nonresponse | 34 | 1 | 10.7039825 | 6.7731864 | 475 | 221 |
| pat79 | SRR2753064 | response | 405 | 1 | 5.24467175 | 13.6509582 | 78 | 41 |
| pat25 | SRR2755094 | nonresponse | 67 | 1 | 1.873303615 | 1.164279571 | 177 | 77 |
| pat90 | SRR2757338 | response | 669 | 0 | 9.977960615 | 56.0915435 | 188 | 91 |
| pat119 | SRR2770872 | long-survival | 108 | 1 | 1.989079222 | 1.583477667 | 717 | 383 |
| pat14 | SRR2771207 | nonresponse | 84 | 1 | 2.541647643 | 1.068664 | 160 | 62 |
| pat43 | SRR2771286 | nonresponse | 37 | 1 | 5.305263077 | 9.6622042 | 401 | 189 |
| pat27 | SRR2771617 | long-survival | 139 | 1 | 3.0778785 | 0.720979167 | 427 | 218 |
| pat47 | SRR2771707 | response | 1106 | 0 | 1.6273971 | 1.378913333 | 24 | 15 |
| pat118 | SRR2772780 | nonresponse | 120 | 1 | 3.136514154 | 2.2977846 | 1624 | 775 |
| pat19 | SRR2774182 | nonresponse | 21 | 1 | 3.504239071 | 3.452168167 | 66 | 39 |
| pat06 | SRR2774280 | nonresponse | 77 | 1 | 6.448114714 | 4.135997 | 240 | 101 |
| pat86 | SRR2775133 | nonresponse | 153 | 1 | 0.8344701 | 1.082288857 | 90 | 55 |
| pat45 | SRR2777065 | nonresponse | 61 | 1 | 0.9939548 | 0.569001667 | 34 | 23 |
| pat46 | SRR2777217 | nonresponse | 36 | 1 | 3.9893123 | 5.813026333 | 34 | 16 |
| pat36 | SRR2777976 | nonresponse | 41 | 1 | 0.3468135 | 2.2696565 | 113 | 57 |
| pat38 | SRR2778056 | response | 302 | 1 | 5.911898786 | 10.78316257 | 256 | 139 |
| pat49 | SRR2778078 | response | 163 | 1 | 8.675801214 | 11.17732714 | 137 | 80 |
| pat08 | SRR2778361 | nonresponse | 73 | 1 | 2.0195744 | 3.088751571 | 191 | 103 |
| pat37 | SRR2778466 | nonresponse | 23 | 1 | 1.250651667 | 1.362827429 | 357 | 164 |
| pat83 | SRR2778609 | long-survival | 107 | 1 | 3.942902857 | 4.913615333 | 171 | 86 |
| pat33 | SRR2779097 | nonresponse | 211 | 1 | 0.71460025 | 0.2752435 | 143 | 52 |
| pat85 | SRR2779183 | nonresponse | 83 | 1 | 10.87537186 | 15.8487985 | 96 | 46 |
| pat123 | SRR2779596 | response | 544 | 1 | 0.9505074 | 1.845861 | 496 | 287 |
| pat44 | SRR2780123 | nonresponse | 62 | 1 | 0.374012 | 1.099694 | 161 | 95 |
| pat04 | SRR2780275 | response | 646 | 0 | 1.891322538 | 3.4458138 | 70 | 37 |
| pat16 | SRR2780299 | long-survival | 84 | 1 | 4.052288231 | 2.351721667 | 28 | 16 |
| pat98 | SRR3083781 | nonresponse | 77 | 1 | 1.252491692 | 1.988774167 | 76 | 45 |

| Patient | AS_Score | Somatic_N | Somatic_HIN | Somatic_Score | CD8A | GZMA | PRF1 | TGFB1 |
| --- | --- | --- | --- | --- | --- | --- | --- | --- |
| pat28 | 3.101476175 | 282 | 148 | 6.64909512 | 4.804274102 | 3.365332967 | 3.86552501 | 3.488603196 |
| pat81 | 7.359508555 | 36 | 26 | 0.283532183 | -1.489592838 | -1.426708418 | -2.03342951 | 4.886240223 |
| pat39 | 151.5413169 | 58 | 29 | 2.102849024 | 3.78460734 | 2.783972748 | 2.390933508 | 3.956336429 |
| pat15 | 13.61235165 | 212 | 104 | 3.68665926 | -2.807014878 | -19.93156857 | -2.766575638 | 4.151309581 |
| pat80 | 2.011443039 | 140 | 97 | 6.774779005 | 2.972426587 | 1.808770677 | 1.765768922 | 4.192931061 |
| pat126 | 8.052356797 | 212 | 100 | 4.122841449 | 2.21866041 | 2.025184391 | 0.340234514 | 2.810874246 |
| pat29 | 4.152977498 | 36 | 29 | 1.426322845 | 1.288440614 | 1.988927977 | 1.703519771 | 3.974640224 |
| pat02 | 24.62500958 | 98 | 63 | 2.823180044 | 3.305452985 | 2.842165379 | 2.846004164 | 5.683138254 |
| pat03 | 35.94732073 | 167 | 78 | 3.25748047 | 0.016574317 | -0.38191568 | -0.647257962 | 3.619908108 |
| pat50 | 13.18804722 | 313 | 184 | 6.033413362 | 1.858644095 | 0.209166887 | -0.368642926 | 3.660233176 |
| pat88 | 19.71452467 | 657 | 343 | 9.473772034 | 5.66829142 | 4.711918351 | 3.799225599 | 3.304299604 |
| pat40 | 27.74990878 | 10 | 6 | 0.090080713 | 1.956049104 | 1.121816762 | -0.926609728 | 4.291114444 |
| pat79 | 14.54810946 | 165 | 111 | 3.408852862 | 1.84265975 | 1.399023629 | 0.856655501 | 2.341194203 |
| pat25 | 12.37556295 | 55 | 33 | 1.489088095 | 2.311806725 | 0.826193713 | 1.263234651 | 2.502437442 |
| pat90 | 10.82280269 | 137 | 54 | 2.646432974 | 2.296093695 | 1.726845867 | 0.814655289 | 4.000538803 |
| pat119 | 49.34580108 | 155 | 81 | 4.090674292 | 1.830303606 | 1.761353208 | -0.453253097 | 1.261878475 |
| pat14 | 11.9452752 | 23 | 17 | 1.14904538 | 2.017766249 | 1.374626077 | 1.053583789 | 5.685169038 |
| pat43 | 41.27345629 | 59 | 35 | 1.182838938 | 5.210311794 | 5.472428381 | 2.959726042 | 4.684456854 |
| pat27 | 14.9927715 | 20 | 11 | 0.739887688 | 1.689471532 | 1.150795174 | 0.199666248 | 4.580684815 |
| pat47 | 0.559005967 | 105 | 82 | 4.023363461 | -0.3293909 | -0.78528673 | -1.485391743 | 4.112892587 |
| pat118 | 100.5854448 | 43 | 20 | 1.089946106 | 3.879623591 | 3.948401246 | 1.674586393 | 3.673778046 |
| pat19 | 5.491824339 | 270 | 153 | 7.938061491 | 3.71345678 | 3.337014512 | 2.479989867 | 4.541163627 |
| pat06 | 9.419974625 | 96 | 70 | 5.778050147 | 4.107838522 | 2.760740352 | 2.84069066 | 4.843760349 |
| pat86 | 3.159782443 | 38 | 19 | 0.70937014 | 1.64883208 | 0.945026255 | 0.902852229 | 3.872964001 |
| pat45 | 1.83521232 | 499 | 272 | 12.10965795 | -5.569391007 | -4.728474641 | -6.228212643 | 3.782476838 |
| pat46 | 0.940049693 | 207 | 102 | 2.146310509 | 0.39755356 | 0.659915082 | -0.284502604 | 5.236435214 |
| pat36 | 5.456661911 | 4 | 0 | 2.91E-11 | -2.95815614 | -0.425258805 | -1.727124505 | 3.475451381 |
| pat38 | 22.68269024 | 1160 | 605 | 32.46437318 | 5.443975374 | 4.682332738 | 4.188253622 | 4.494664634 |
| pat49 | 10.16610518 | 260 | 137 | 4.052453818 | 6.136961544 | 4.244958336 | 4.836882525 | 4.658829364 |
| pat08 | 14.40169054 | 238 | 125 | 4.865998694 | 3.151400585 | 1.96179413 | 1.943424944 | 3.165444943 |
| pat37 | 23.87057516 | 81 | 57 | 2.819788472 | 2.807120953 | 1.91054251 | 0.321801636 | 3.436071918 |
| pat83 | 6.884499879 | 14 | 9 | 0.175221788 | 4.603645839 | 3.562177 | 2.803108839 | 3.769991265 |
| pat33 | 5.164238204 | 10 | 6 | 0.845077167 | -0.57584419 | -0.750540524 | -1.296889909 | 3.125122061 |
| pat85 | 10.68025956 | 74 | 39 | 2.054132098 | 6.386388014 | 4.876427126 | 4.265292005 | 5.569291512 |
| pat123 | 34.70006798 | 300 | 182 | 7.343790919 | 1.649213357 | 0.58627 | -0.941880731 | 2.43052648 |
| pat44 | 15.70510487 | 26 | 13 | 0.628411601 | -4.487300956 | -5.590872534 | -4.631282612 | 3.232637935 |
| pat04 | 4.891851252 | 209 | 105 | 4.760680108 | 2.823469485 | 2.633029801 | 2.847092286 | 4.699814538 |
| pat16 | 0.018421264 | 806 | 466 | 19.36487474 | 2.990090614 | 2.702660672 | 1.194029314 | 2.166605303 |
| pat98 | 5.605079518 | 15 | 9 | 0.368217974 | 2.748779892 | 1.690686028 | 0.72621027 | 3.306093918 |
