# Supplementary material for: ASNEO: Identification of personalized alternative splicing based neoantigens with RNA-seq
Source: Aging (Albany NY). 2020 Jul 22;12(14):14633–48. doi: 10.18632/aging.103516 (PMC7425491; doi:10.18632/aging.103516)
Supplement: Supplementary Table 2 [file aging-12-103516-s001..docx]

**Supplementary Table 2. Information about 25 patients used in Hugo cohort.**

| Patient | Sample | Response | OS | Event | T cell | CTL | AS_N | AS_HIN |
| --- | --- | --- | --- | --- | --- | --- | --- | --- |
| Pt1 | SRR3184279 | PD | 607 | 1 | 4.765398357 | 5.5298855 | 56 | 27 |
| Pt2 | SRR3184280 | PR | 927 | 0 | 18.17112967 | 3.0630368 | 57 | 26 |
| Pt4 | SRR3184281 | PR | 948 | 0 | 16.74625621 | 23.10599117 | 87 | 51 |
| Pt5 | SRR3184282 | PR | 439 | 0 | 10.53196411 | 7.07785225 | 90 | 47 |
| Pt6 | SRR3184283 | PR | 882 | 0 | 3.7266161 | 2.601708833 | 64 | 44 |
| Pt7 | SRR3184284 | PD | 662 | 1 | 66.67385922 | 31.22168417 | 8 | 7 |
| Pt9 | SRR3184286 | CR | 1054 | 0 | 13.10422686 | 2.021252857 | 67 | 37 |
| Pt10 | SRR3184287 | PD | 387 | 0 | 7.972937 | 1.852676 | 56 | 25 |
| Pt12 | SRR3184288 | PD | 327 | 1 | 7.598492889 | 6.7979942 | 45 | 20 |
| Pt13 | SRR3184289 | CR | 917 | 0 | 2.682193571 | 7.983649286 | 68 | 44 |
| Pt14 | SRR3184290 | PD | 54 | 0 | 5.228492286 | 2.822469571 | 45 | 28 |
| Pt15 | SRR3184291 | PR | 980 | 1 | 9.847678714 | 3.846483 | 5 | 1 |
| Pt16 | SRR3184292 | PD | 186 | 1 | 7.383498429 | 3.010828 | 23 | 12 |
| Pt19 | SRR3184293 | PR | 1060 | 0 | 6.647484909 | 2.666862333 | 61 | 31 |
| Pt20 | SRR3184294 | PD | 337 | 1 | 9.005329733 | 28.10481029 | 33 | 14 |
| Pt22 | SRR3184295 | PD | 182 | 1 | 7.881365533 | 3.531568714 | 67 | 24 |
| Pt23 | SRR3184296 | PD | 103 | 1 | 3.725355214 | 4.542355143 | 15 | 10 |
| Pt25 | SRR3184297 | PD | 262 | 1 | 6.871396929 | 4.677147667 | 19 | 9 |
| Pt28 | SRR3184300 | PR | 439 | 1 | 7.468688857 | 7.185576 | 121 | 51 |
| Pt29 | SRR3184301 | PD | 269 | 1 | 8.021107333 | 4.608647143 | 60 | 38 |
| Pt31 | SRR3184302 | PD | 704 | 0 | 4.5263633 | 4.136256 | 22 | 15 |
| Pt32 | SRR3184303 | PD | 171 | 1 | 4.8458604 | 4.905503667 | 12 | 8 |
| Pt35 | SRR3184304 | PR | 427 | 0 | 6.712201143 | 6.361892286 | 75 | 34 |
| Pt37 | SRR3184305 | PR | 364 | 0 | 4.0497375 | 23.05943833 | 17 | 6 |
| Pt38 | SRR3184306 | PR | 448 | 0 | 23.1530378 | 8.796858667 | 16 | 12 |

| Patient | AS_Score | Somatic_N | Somatic_HIN | Somatic_Score | CD8A | GZMA | PRF1 | TGFB1 |
| --- | --- | --- | --- | --- | --- | --- | --- | --- |
| Pt1 | 3.806105132 | 674 | 387 | 18.09522801 | 2.35385112 | 3.312851364 | 1.95748489 | 4.711389441 |
| Pt2 | 3.437740752 | 932 | 541 | 26.67710818 | 2.403227343 | 2.638351951 | 0.516404763 | 4.397365319 |
| Pt4 | 10.78663845 | 1733 | 1057 | 48.1728738 | 2.303630841 | 2.223543782 | 1.756872375 | 3.130471931 |
| Pt5 | 5.655652077 | 224 | 126 | 7.24106611 | -2.164504666 | -2.367648117 | -1.671061671 | 4.42931078 |
| Pt6 | 9.424259579 | 80 | 60 | 3.517131049 | -1.174703382 | -0.630730364 | 0.569900176 | 4.972314021 |
| Pt7 | 0.012485244 | 438 | 222 | 9.489866148 | 0.994433477 | 1.371507761 | 0.193628644 | 4.137272502 |
| Pt9 | 3.586333781 | 154 | 104 | 3.718908036 | 2.985233923 | 3.896564902 | 1.776501479 | 4.417288928 |
| Pt10 | 2.16388065 | 92 | 63 | 4.331952853 | -0.438593818 | -1.505712008 | -1.146564708 | 4.894461241 |
| Pt12 | 4.776492467 | 11 | 10 | 0.00257598 | -1.813642323 | 1.320864526 | 0.285460793 | 4.010665245 |
| Pt13 | 7.066437393 | 206 | 99 | 5.90177196 | 4.081259821 | 4.063018135 | 2.627371049 | 5.448053066 |
| Pt14 | 6.082954934 | 799 | 497 | 20.61033837 | 1.267597859 | 2.770172329 | 1.577845069 | 5.588906066 |
| Pt15 | 0.474888406 | 248 | 131 | 6.700164668 | 2.705103548 | 3.078284063 | 1.542606515 | 2.371649381 |
| Pt16 | 0.988137771 | 111 | 67 | 3.423957341 | 0.530394369 | 0.560110615 | 2.020490897 | 4.190612712 |
| Pt19 | 5.584151018 | 233 | 144 | 7.053403384 | 2.058205566 | 1.964878568 | 1.419267133 | 6.436139713 |
| Pt20 | 4.28055167 | 161 | 93 | 3.107838517 | 5.889197583 | 6.325436754 | 4.671454365 | 3.686353247 |
| Pt22 | 5.82039674 | 13 | 1 | 0.170555467 | 3.366096739 | 3.861386834 | 2.156564497 | 5.195574766 |
| Pt23 | 2.109206755 | 67 | 33 | 1.364241837 | 3.618204397 | 3.543124941 | 3.015862493 | 4.248654201 |
| Pt25 | 2.82712443 | 110 | 60 | 2.908516882 | 3.892603062 | 4.48919905 | 3.513917843 | 3.468409457 |
| Pt28 | 12.25997468 | 137 | 59 | 1.58856173 | -1.184343292 | 0.480897204 | -0.702435734 | 3.974710155 |
| Pt29 | 9.643129381 | 78 | 43 | 1.775604993 | 3.815637302 | 4.248013393 | 1.809403375 | 3.797118611 |
| Pt31 | 6.503905235 | 300 | 155 | 5.977355367 | 0.934099084 | 1.066126346 | 2.816096255 | 5.362960238 |
| Pt32 | 0.270307265 | 207 | 108 | 3.888709424 | 0.410140118 | 3.288929025 | 0.001851726 | 6.025352311 |
| Pt35 | 3.698068445 | 162 | 86 | 3.577039482 | 2.636525271 | 2.312851178 | 1.519912644 | 4.032157739 |
| Pt37 | 1.40090636 | 164 | 105 | 5.707444526 | 2.469039065 | 2.866896117 | 1.556493875 | 2.677015774 |
| Pt38 | 0.03355981 | 9 | 6 | 0.131944277 | 4.011015022 | 4.048539339 | 3.350847313 | 5.577001211 |

*Note:*

| PFS | Progress free survival |
| --- | --- |
| OS | Overall survival |
| PD | Progressive Disease |
| PR | Partial Response |
| CR | Complete Response |
| T cell | The abundance of T cell calculated by MCPcounter |
| CTL | The abundance of cytotoxic lymphocyte (CTL) calculated by MCPcounter |
| AS_N | AS neopeptide number |
| AS_HIN | AS high immunogenicity neopeptide number |
| AS_Score | The total immune score for all AS neopeptides in a sample |
| Somatic_N | Somatic neopeptide number |
| Somatic_HIN | Somatic high immunogenicity neopeptide number |
| Somatic_Score | The total immune score for all somatic neopeptides in a sample |
| CD8A | The log expression level (RPKM) of gene CD8A |
| GZMA | The log expression level (RPKM) of gene GZMA |
| PRF1 | The log expression level (RPKM) of gene PRF1 |
| TGFB1 | The log expression level (RPKM) of gene TGFB1 |
