# Supplementary material for: ASNEO: Identification of personalized alternative splicing based neoantigens with RNA-seq
Source: Aging (Albany NY). 2020 Jul 22;12(14):14633–48. doi: 10.18632/aging.103516 (PMC7425491; doi:10.18632/aging.103516)
Supplement: Supplementary Table 3 [file aging-12-103516-s003..pdf]

SUPPLEMENTARY TABLE

Supplementary Table 3. Univariate and multivariate cox regression.

Variable	van Allen cohort						Hugo cohort					
	Univariate analysis			Multivariate analysis (gender+age)			Univariate analysis			Multivariate analysis (gender+age)		
	HR	95% CI	P	HR	95% CI	P	HR	95% CI	P	HR	95% CI	P
Burden*CTL*Tcell	0.45	0.20-1.01	0.05	0.41	0.18-0.93	0.03	-	-	-	-	-	-
HIN*CTL*Tcell	0.42	0.18-0.98	0.04	0.40	0.17-0.94	0.04	-	-	-	-	-	-
Burden*CTL*Tcell	-	-	-	-	-	-	0.30	0.09-0.99	0.04	0.26	0.08-0.89	0.03
HIN	-	-	-	-	-	-	0.21	0.06-0.76	0.009	0.18	0.05-0.68	0.01
